# Phase dependent hypothalamic activation following trigeminal input in cluster headache

**DOI:** 10.1186/s10194-020-01098-2

**Published:** 2020-03-30

**Authors:** Laura H. Schulte, Ame Abdu Haji, Arne May

**Affiliations:** 1 Department of Systems Neuroscience, University Medical Center Eppendorf, University of Hamburg, Hamburg, Germany; 2 Clinic for Psychiatry, University Medical Center Eppendorf, University of Hamburg, Hamburg, Germany

**Keywords:** Cluster headache, Hypothalamus, Brainstem fMRI, Nociceptive stimulation

## Abstract

**Background:**

Task-free imaging approaches using PET have shown the posterior hypothalamus to be specifically activated during but not outside cluster headache attacks. Evidence from task related functional imaging approaches however is scarce.

**Methods:**

Twenty-one inactive cluster headache patients (episodic cluster headache out of bout), 16 active cluster headache patients (10 episodic cluster headache in bout, 6 chronic cluster headache) and 18 control participants underwent high resolution brainstem functional magnetic resonance imaging of trigeminal nociception using gaseous ammonia as a painful stimulus.

**Results:**

Following trigeminonociceptive stimulation with ammonia there was a significantly stronger activation within the posterior hypothalamus in episodic cluster headache patients out of bout when compared to controls. When contrasting estimates of the pain contrast, active cluster headache patients where in between the two other groups but did not differ significantly from either.

**Conclusion:**

The posterior hypothalamus might thus be hyperexcitable in cluster headache patients outside the bout while excitability to external nociceptive stimuli decreases during in bout periods, probably due to frequent hypothalamic activation and possible neurotransmitter exhaustion during cluster attacks.

## Introduction

In most *Task-free* imaging approaches like H_2_O-PET and restingstate fMRI, the posterior hypothalamus has been shown to be specifically activated during but not outside cluster headache attacks with activity levels normalising after pain relieve due to sumatriptan administration [1–3]. Furthermore, hypothalamic restingstate connectivity is altered in cluster headache patients as compared to healthy controls [4, 5] thus suggesting a crucial role of this brain area in the pathophysiology of cluster headache Evidence from *Task-related* functional imaging approaches however is scarce – to our knowledge there are to date no stimulation fMRI studies in cluster headache patients although investigating the trigeminal nociceptive functioning in active and inactive cluster headache might add important information to our current understanding of cluster headache pathophysiology. There is however some evidence of altered nociceptive and autonomic functioning in cluster headache: temporal nociceptive processing seems to be facilitated during the active cluster headache phases while markers of supraspinal pain control might be defective [6]. Accordingly, in cluster headache patients there is evidence for trigeminonociceptive facilitation on brainstem level [7]. Here we hypothesized that hypothalamic processing of trigeminal nociceptive stimuli might be altered in cluster headache patients as compared to healthy controls. We investigate active and non-active cluster headache patients using a well-established paradigm for functional magnetic resonance imaging of trigeminal nociception.

## Methods

### Participants

Cluster headache patients were recruited via the Headache Outpatient Department of the University Medical Center Eppendorf in Hamburg, Germany, and a local database between August 2014 and November 2016. Patients were divided into 2 groups: active cluster headache (including both episodic cluster headache patients in bout and chronic cluster headache patients) and inactive cluster headache (episodic cluster headache patients out of bout). Three patients participated both in the active state and in the inactive state. Control participants did not suffer from any severe neurological or internal illness and had no history of pain or headache diseases including migraine, cluster headache and frequent tension type headache. Written informed consent was obtained from all participants.

### Experimental paradigm

Participants underwent one session of event-related fMRI using a previously established protocol of standardized nociceptive stimulation of the nasal mucosa using gaseous ammonia [8–13]. Additionally, 3 of the episodic cluster headache patients underwent a second identical session within bout period. Each session consisted of three parts, during which 4 different stimuli were presented in pseudorandomized order ensuring no two identical stimuli directly followed each other. Each stimulus was preceded by a reaction task and followed by a rating procedure during which intensity and unpleasantness of the respective stimulus had to be rated. The 4 stimuli were gaseous ammonia as an activator of trigeminonociceptive afferents within the nasal mucosa, rose odour as a purely olfactory stimulus, air puffs as a control condition and a rotating checkerboard as visual stimulus. The gaseous stimuli were presented via a Teflon tube within the participants’ nostril (in case of cluster headache patients on the site of the cluster pain) using a custom built olfactometer. The visual stimulus was presented via a beamer-mirror system that was also used for presentation of the reaction task and the rating procedure. Presentation software was used for stimulus presentation, timing and logging of experimental data.

### Image acquisition

MRI images were acquired on a 3 T scanner (Siemens TIM TRIO) using a 32-channel head coil. All participants lay supine on the back in the scanner. Echoplanar images were recorded via a protocol optimized for high resolution brainstem imaging (voxel sizes 1.25 × 1.25 × 2.5 mm^3^, TR 2.61 s, TE 27 ms, 38 axial slices, FoV 216 × 108 mm^2^, matrix: 172 × 86, flip angle 80°, GRAPPA-accelerated). High resolution T1 weighted images were acquired using an MPRAGE sequence (voxel size 1 × 1 × 1 mm^3^, TR 2.3 s, TE 2.98 ms, FoV 192 × 256 × 240 mm^3^, slice orientation: sagittal, flip angle 9°, inversion time 1.1 s).

### Preprocessing

Preprocessing was performed using SPM 12. Preprocessing steps included slice timing, image realignment, spatial normalization and warping into MNI space via a segmentation-normalization sequence consisting of co-registration of the structural images to the mean functional image, segmentation of the structural images and normalisation of functional images using segmentation parameters of the structural image of each subject. After normalization, images were smoothed using a 4 mm full width at half maximum Gaussian kernel.

### Flipping

Cluster headache patients were stimulated on the site of the cluster. Accordingly, to make the stimulation site homonymous, images of the right sided cluster-headache patients were left-right flipped to match the stimulation site of the other patients using the in-built function fslswapdim of FSL.

### Physiological denoising

Heart- and breathing rate were recorded via a respiratory belt and pulsoxymetry and included in the first level analysis as nuisance regressors using the selective averaging method of Deckers et al., that has in detail been described elsewhere [14].

### Statistical analysis

#### 1st level

Statistical analysis was done using SPM12 and Matlab version R2014b. First level general linear model included 3 sessions with 4 experimental regressors: ammonia, rose odour, air and checkerboard stimulation. Button presses, anticipation phase, 12 movement regressors (realignment parameters) and 18–20 physiological regressors of cardiac and breathing state were included as nuisance regressors. In case of the gaseous stimuli, a delta function at event-onset was convolved with the hemodynamic response function. The checkerboard was modelled using a box-car function with a duration of 4 s.

#### 2nd level

For the second level analysis, the first level pain-contrast images (ammonia > air) of the individual participants were entered into an Anova consisting of the following groups: inactive cluster headache (out of bout), active cluster headache (ECH in bout and CCH) and healthy controls. Results were generally regarded significant at *p* < 0.05, corrected for multiple comparisons using the family wise error rate (FWE). As the posterior hypothalamus was a predefined region of interest, we performed a small volume correction using a 6 mm sphere around the peak coordinates reported for the posterior hypothalamus by Maniyar et al. transferred to the left side (− 6, − 6, − 12) [15], to which FWE-correction was restricted.

## Results

**Fig. 1:**
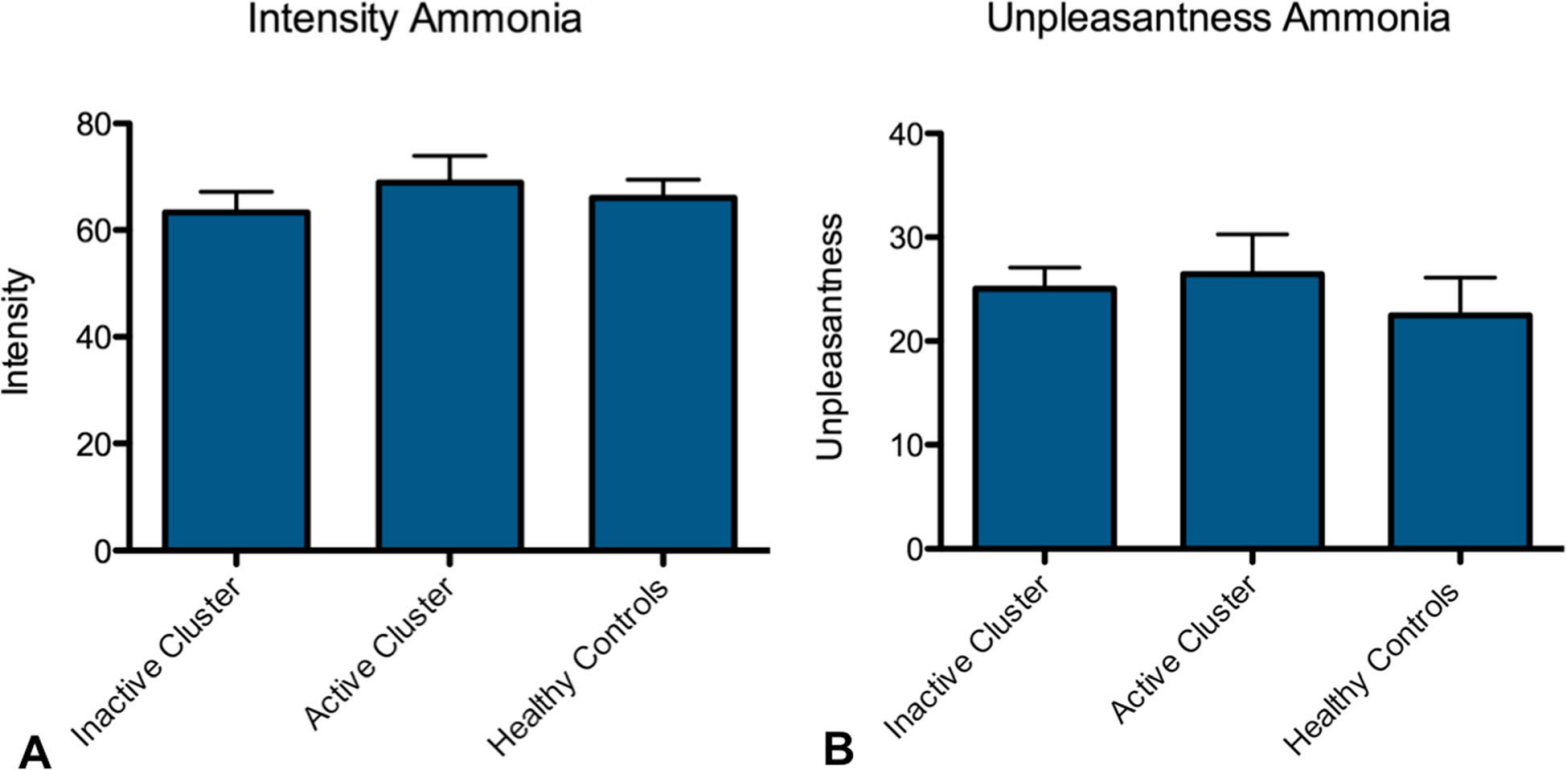
Intensity and unpleasantness ratings of the ammonia stimulus among the three study groups. Bars depict the mean within each group. Error bars indicate standard error of mean. No significant differences were observed regarding intensity and unpleasantness ratings between the three study groups

Fifty-five participants where included in the final analysis (21 episodic cluster headache patients out of bout, 16 active cluster headache patients (10 eCH in bout, 6 CCH) and 18 control participants). Three episodic cluster headache patients were scanned twice: in- and outside the active period. Among the active group, 9 patients took prophylactic medication balanced by 6 patients within the inactive group. Details can be found in Table 1. Statistical analysis of the intensity and unpleasantness ratings of the ammonia-stimulus revealed no statistically significant differences between the three groups. A plot of intensity and unpleasantness of the ammonia stimulus can be found in Fig. 1.

**Table 1:** Characteristics of the investigated patients groups

	Age	Sex	Years of cluster headache	Number of patients taking prophylactic medication	Medication taken
Active Cluster Headache	50,0833333	12 m, 4 f	13,203,125	9	Verapamil (2), Verapamil+Topiramate (1), Lithium (2), Prednisolon (1), Allegro (1), Verapamil+Allegro (1), Almotriptan (1)
Inactive Cluster Headache	45,2,380,952	18 m, 3 f	14,55	6	Verapamil (3),Topiramate (1), Verapamil + Topiramate (2)

**Fig. 2:**
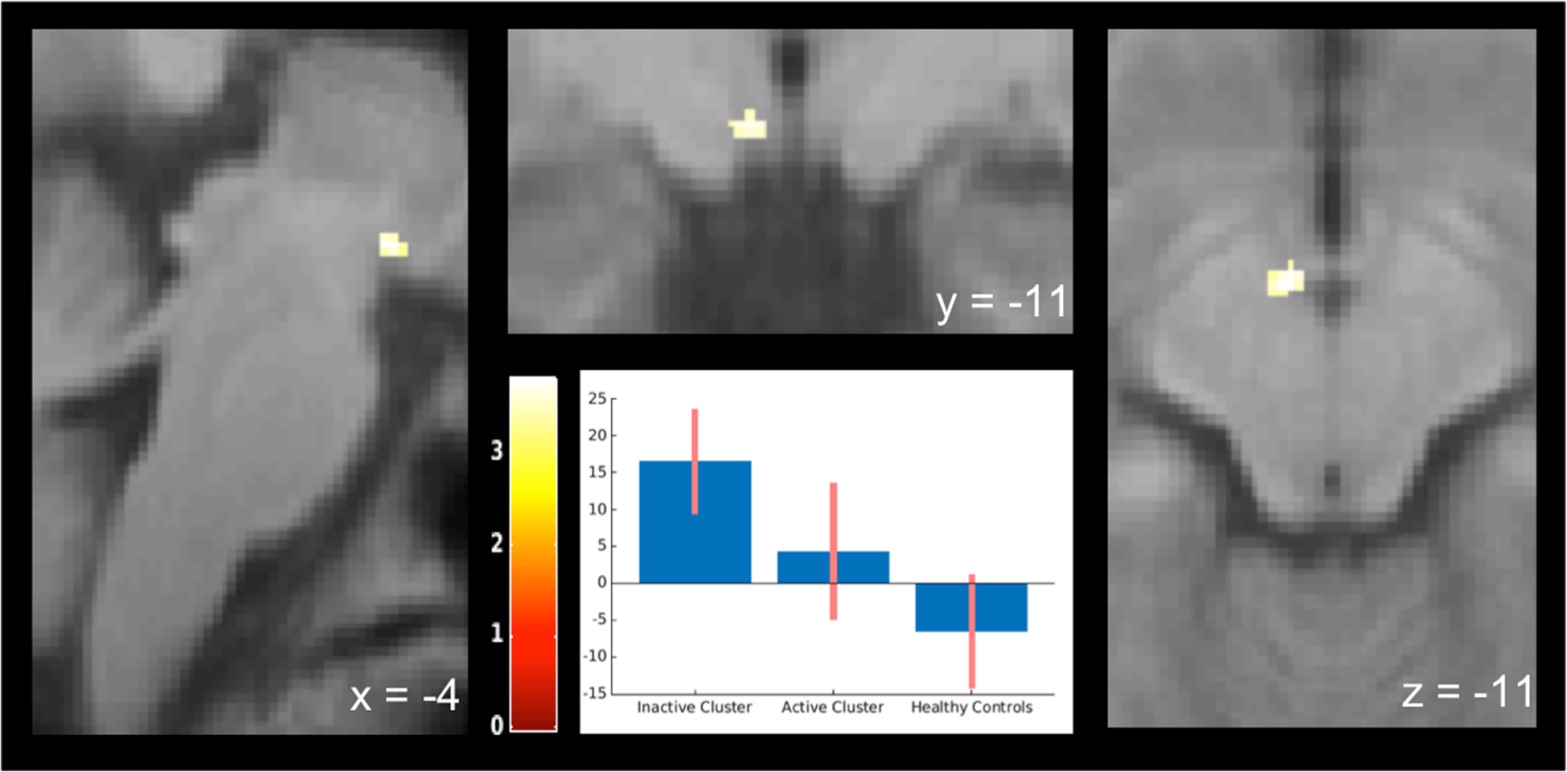
T-score map of the second level anova, contrast inactive cluster headache > controls. Visualization threshold *p* < 0.001. Significant activation (*p* < 0.05, small-volume-FWE-corrected) was observed within the posterior hypothalamus ipsilateral to the site of cluster headache and trigeminal nociceptive stimulation (x = − 4, y = − 11, z = − 11, T = 3.80, k = 12 voxels). The bar plot indicates contrast estimates and 95%-confidential intervals for the three groups at the maximum of posterior hypothalamic activation

Following trigeminonociceptive stimulation with ammonia (ammonia > air), we found in the functional imaging data a significantly stronger activation within the posterior hypothalamus (x = − 4, y = − 11, z = − 11, T = 3.80, *p* < 0.05, small-volume-FWE-corrected) in episodic cluster headache patients out of bout when compared to controls. When looking at the contrast estimates of the pain contrast, active cluster headache patients (CCH and ECH in bout) where in between the two other groups but did not differ significantly from either. Figure 2 shows a T-score map of the contrast eCH out of bout vs. controls and a plot of contrast estimates of the 3 groups.

## Discussion

The posterior part of the hypothalamus has been shown to be activated during acute cluster headache attacks in various studies, starting with early H_2_O-PET studies and continuing to recent fMRI studies [1–3, 16–18]. To date, no studies exist which investigated cluster headache patients using task related designs in functional imaging. Our data of 37 patients using trigeminal nociceptive input as a task (but outside the cluster headache attack) corroborate hypothalamic involvement in cluster headache pathophysiology. We note that trigeminal nociceptive input activates the posterior part of the hypothalamus, a brain area which is associated with cluster headache pathophysiology [19], and not the hypothalamus proper. Whilst it is undoubted that it is indeed crucial for cluster headache pathophysiology [20], the correct anatomical denomination is a much discussed issue [21–23]. For the sake of convenience we keep the wording of the original report [2] and consistently refer to this area as posterior hypothalamus. Regarding the site of activation, our data are in line with previous studies showing the activation ipsilateral to the site of cluster headache [2, 16]. However, different from what we expected, hypothalamic activity levels following trigeminal nociceptive stimulation were highest in patients out of bout, i.e. with inactive cluster, and not within the active group. As there were patients taking medication within both cluster groups, it is rather unlikely that the difference we describe is simply due to an effect of medication. The hypothalamus is involved in processing and modulation of painful trigeminal stimuli via various fiber connections of different hypothalamic nuclei to the spinal trigeminal nucleus, the PAG, the posterior Raphe nucleus and others [10, 18]. Additionally, there are efferent fiber connections of the spinal trigeminal nucleus that directly activate hypothalamic nuclei [19] and the hypothalamus has been shown to be activated in response to trigeminonociceptive stimulation in healthy subjects [10]. It is thus strategically positioned for integrating painful stimuli with signals of energy homeostasis and circadian rhythmicity. Cyclic activity changes within the hypothalamus might constitute the neuronal basis for the beginning of the cluster bout and especially the initiation of a cluster headache attack [24]. Since the posterior hypothalamus is thus frequently activated in active cluster headache (eCH in bout and cCH), these frequent episodes of activation might lead to neurotransmitter exhaustion and hypo-excitability of this area to external stimuli. To our knowledge there are to date no studies directly investigating hypothalamic responses to external stimuli in cluster headache patient. One behavioral measure often discussed to be influenced by hypothalamic activity is the nociceptive blink reflex. Studies point towards altered habituation of the blink reflex in cluster headache patients in the bout and in one study also out of bout [7, 25, 26], but findings are not homogeneous and partly contradicting [27]. Hypothalamic involvement in this mechanism is therefore likely but not proven. The alterations in blink reflex habituation might thus point towards altered hypothalamic functioning in cluster headache but can not help us to explain the current results since other trigeminonociceptive sites of conduction might be involved in mediating blink reflex habituation. The theory of a hypothalamic exhaustion and hypoexcitability in active cluster headache is thus a viable explanation although there is currently no direct experimental evidence to support it. It could account for the fact that, other than expected, hypothalamic activation following nociceptive stimulation of the nasal mucosa was not highest within the active cluster group but within the group of inactive episodic cluster headache patients.

## Conclusions

Our data suggest that the posterior hypothalamus might be hyperexcitable in cluster headache patients but only outside the bout while excitability (to external nociceptive stimuli) decreases during in bout periods, possibly due to frequent hypothalamic activation during cluster attacks.

## Data Availability

The datasets generated and analysed during the current study are not publicly available due to national data protection acts. Completed analyses might be made available from the corresponding author on reasonable request.
